# CARTA fellows’ scientific contribution to the African public and population Health Research agenda (2011 to 2018)

**DOI:** 10.1186/s12889-020-09147-w

**Published:** 2020-06-29

**Authors:** Jude O. Igumbor, Edna N. Bosire, Tariro J. Basera, Dieudonne Uwizeye, Olufunke Fayehun, Hesborn Wao, Ademola Ajuwon, Emmanuel Otukpa, Florah Karimi, Daphney Conco, Evelyn Gitau, Sharon Fonn

**Affiliations:** 1 School of Public Health, Faculty of Health Sciences, University of the Witwatersrand, Johannesburg, South Africa; 2 South African Medical Research Council Developmental Pathways for Health Research Unit (DPHRU), School of Clinical Medicine, Faculty of Health Sciences, University of the Witwatersrand, 27 St Andrews Road, Parktown, Johannesburg, South Africa; 3 School of Governance, University of Rwanda, Kigali, Rwanda; 4 Department of Sociology, University of Ibadan, Ibadan, Nigeria; 5 African Population and Health Research Centre, Nairobi, Kenya; 6 Department of Health Promotion and Education, University of Ibadan, Ibadan, Nigeria

**Keywords:** Population and health priorities, Doctoral research, African agenda, Peer review publications

## Abstract

**Background:**

Since its inception in 2009, the Consortium for Advanced Research Training in Africa (CARTA) program has focused on strengthening the capacity of nine African universities and four research centres to produce skilled researchers and scholars able to improve public and population health on the continent. This study describes the alignment between CARTA-supported doctoral topics and publications with the priorities articulated by the African public and population health research agenda.

**Methods:**

We reviewed the output from CARTA PhD fellows between 2011 and 2018 to establish the volume and scope of the publications, and the degree to which the research focus coincided with the SDGs, World Bank, and African Development Bank research priority areas. We identified nine key priority areas into which the topics were classified.

**Results:**

In total, 140 CARTA fellows published 806 articles in peer-reviewed journals over the 8 years up to 2018. All the publications considered in this paper had authors affiliated with African universities, 90% of the publications had an African university first author and 41% of the papers have CARTA fellows as the first author. The publications are available in over 6300 online versions and have been cited in over 5500 other publications. About 69% of the published papers addressed the nine African public and population health research agenda and SDG priority areas. Infectious diseases topped the list of publications (26.8%), followed by the health system and policy research (17.6%), maternal and child health (14.7%), sexual and reproductive health (14.3%).

**Conclusions:**

Investments by CARTA in supporting doctoral studies provides fellows with sufficient training and skills to publish their research in fields of public and population health. The number of publications is understandably uneven across Africa’s public and population priority areas. Even while low in number, fellows are publishing in areas such as non-communicable disease, health financing, neglected tropical diseases and environmental health. Violence and injury is perhaps underrepresented. There is need to keep developing research capacity in partner institutions with low research output by training more PhDs in such institutions and by facilitating enabling environments for research.

## Background

Africa has a disproportionate burden of both infectious and non-communicable diseases (NCDs) compared with other world regions [1]. Current disease estimates indicate increases in the incidence of NCDs, including cardiovascular diseases (like hypertension and stroke), cancers, and diabetes, which are now major causes of morbidity and mortality and are projected to overtake infectious diseases by 2030 [2]. This is occurring when most African countries are still struggling to control infectious diseases – such as Human Immunodeficiency Virus and Acquired Immunodeficiency Syndrome (HIV and AIDS) and Tuberculosis (TB), due to weak and overburdened health systems [3]; inadequate resources for scaling up proven interventions; poor management of human resources for health; and recurrent natural and man-made disasters and emergencies [4]. Other contributors to the heavy disease burden on the continent include food insecurity, poor access to safe sanitation, the prevalence of indoor pollutants, increased rates of unemployment, violence and forced migration and access to cheap but unhealthy foods [5]. Tackling these challenges and coming up with pragmatic solutions requires robust research [6, 7].

This need for local research is occurring when Africa has in the recent past been reported to lag behind other regions in research output – producing less than 1% of the world’s research [8], including research in the fields of population and health [9, 10]. The under-performance in research in Africa is partly due to inadequate research funding by African governments [11, 12]. During the first African Ministerial Conference on Science and Technology in 2003, participating countries committed to spending at least 1% of their gross domestic product on research and development by 2010 [12, 13]. Three countries, Malawi, Uganda, and South Africa, had honoured this commitment five years down the line, in 2015 [14]. Other reasons for under-performance include inadequate access to research training [9], poor research infrastructure and technology such as laboratories and computers, insufficient mentorship for junior researchers [11], and limited collaboration or partnerships among research institutions within Africa [10].

There has however been a substantial improvement in the output of researchers in the field of public and population health in many African universities during the past decade [15, 16], a development attributed to advancement in the faculties of public and population health in higher education institutions [16, 17]. Nachega and colleagues [16] reported that between 1991 and 2010, epidemiology and public health research output in the World Health Organization (WHO)/African Region (AFRO) increased from 172 to 1086 peer-reviewed articles per annum, which is a 631% increase over 19 years. During this period, the most commonly research topics published by researchers from African institutions were on HIV/AIDS (11%), malaria (9%), and tuberculosis (7%), which may be motivated by the articulation of the Millennium Development Goals (MDGs) and increased donor funding in specific disease areas [18].

There have been a number of contributions to the development of research capacity on the continent. While not yet adequate, there has been both national and international investment in higher education in general [19, 20]. There has also been a commitment to health-related research. The New African Region Health Research Strategy adopted by the African Union Health ministers in 2015, has prioritised public and population health research [21]. Significant investment in health related research capacity building has also taken place, including the African Institutions Initiative [22], and investments made through the Developing Excellence in Leadership, Training and Science (DELTAS) programme [23], as well as the establishment of the Centre for Disease Control (Africa CDC) by the African Union General Assembly in 2015 [24].

As the disease burden changes on the continent, a comprehensive approach is needed to guide health research capacity in the region, and policies and interventions need to be appropriate to local conditions. The conduct and dissemination of good quality research undertaken by African scientists is central to this [25]. We investigated the research output of a cohort of African scholars to assess the degree to which their research is related to the identified African health priorities.

We focused on one initiative for which we had comprehensive data, the Consortium for Advanced Research Training in Africa (CARTA) [26]. Launched in 2009, CARTA initially brought together nine academic and four research institutions from seven countries in Africa, in partnership with selected non-African universities and training institutes. CARTA aims to develop sustainable health research capacity in Africa through training of PhD fellows in public and population health and promoting research supportive environments. Part of CARTA’s strategy for long term sustainability is that staff of participating African consortium institutions are admitted and supported to obtain their PhD, as a way to build and multiply research capacity in the continent [26]. This study describes the publications authored by CARTA supported PhD fellows and compared them to published consensus on African public and population health research agenda of the sustainable development goals (SDGs), World Bank (WB) and Africa Development Bank (AfDB).

## Methods

### Study design

A bibliometric search of research output from CARTA PhD fellows between 2011 and 2018 was conducted to establish the volume and scope of the publications. In 2011 there were 20 fellows in the programme and by 2018, 185 fellows had been admitted in the fellowship. We did not include any paper published by the fellows before they were recruited into the program. We compared the content of the publications with research priorities identified by the SDGs, WB and AfDB. A list of public and population health priorities identified by SGD, WB, and AfDB was developed to categorise the CARTA fellows’ publication outputs. This process resulted in the nine priority area in which CARTA fellows have published including: (1) infectious diseases, (2) non-communicable diseases (NCDs), (3) mental health, (4) sexual and reproductive health (SRH), (5) maternal and child health (MCH), (6) health systems and policy, (7) violence and injuries, (8) food security and nutrition and (9) environmental health. Other publications were categorised as “others” and included topics in education, demography, capacity building, pharmacology, microbiology, and occupational health.

### Data selection and analysis

A total of 806 publications were received from the CARTA database maintained by the CARTA secretariat which is hosted by the African Population and Health Research Centre (APHRC), Nairobi, Kenya. The publications were validated for the accuracy of citation by checking each publication online through PubMed, Google Scholar, or ResearchGate databases. In cases where the publications were not available in these sources, the respective CARTA fellow were contacted to verify the full citation of their publications. We omitted study publications that were not peer-reviewed, and publications that could neither be accessed online nor verified as accurate citations by the respective authors contacted. We also excluded publications submitted for review before an individual’s CARTA fellowship period because the CARTA intervention would not have contributed to the conceptualization of those specific publications. We further excluded books, theses, editorials, commentaries, and blog publications, as we could not establish whether the publication was a result of a peer-review process.

The study topics were exported into Microsoft Excel for manual validation and classification. For each publication, we also captured the affiliations of the fellows (both the university where they were employed (their home university) and where they are registered for their degree (if this was different from their home institution), the number of online versions of the publication available, and the number of times each publication has been cited in other peer-reviewed publications. We also captured the number of CARTA fellows in each publication, the order of authorships, other funding sources for the studies and the journal’s impact factor. The results are presented as proportions and frequencies using a combination of tables and graphical methods.

The focus or main field of research of each study was categorised and then cross-matched with the SDG, World Bank (WB), and African Development Bank (AfDB) Agenda 2063’s Goal 3 on Healthy and Well-nourished Citizens for Africa as summarised in Table 1. This was done independently by three reviewers and discrepancies in the classification were discussed and verified by a fourth reviewer.

**Table 1:** Summary of health priorities in which CARTA fellows have published

**Health priorities**	**SDG**	**WB**	**AfDB**
1. Infectious diseases	✓	✓	✓
2. Non-communicable diseases (NCDs)	✓		
3. Sexual and reproductive health (SRH)	✓	✓	✓
4. Maternal and child health (MCH)	✓	✓	✓
5. Health Systems and Policy	✓	✓	✓
6. Violence and injuries	✓	✓	
7. Food security and nutrition	✓	✓	✓
8. Mental health and substance abuse	✓	✓	
9. Environmental health	✓	✓	✓

*SDG* Sustainable Development Goals; *WB* World Bank; *AfDB* African Development Bank

## Results

### Publication pattern

**Fig. 1:**
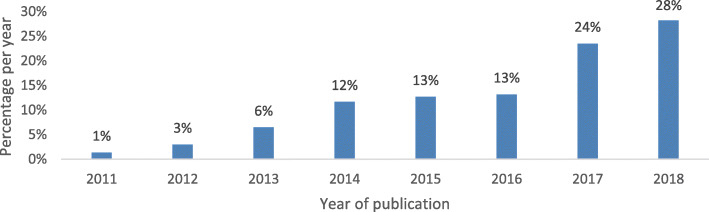
Percentage distribution of all articles published by CARTA fellows from 2011 to 2018 by year of publication (*N* = 806)

In total, 140 CARTA fellows enrolled in CARTA program published 806 articles during the period under review. The annual number of peer review articles published by the fellows increased from 11 articles in 2011 to 223 articles in 2018. Figure 1 shows the increase in the number of articles published over time. The publications were available in 6308 online versions and had been cited in 5529 publications (Table 2). The journals that the fellows published in have a median impact factor of 2.10 (Interquartile range (IQR): 1.04–2.78). Given the nature of the programme and our inclusion criteria, all the publications considered in this paper had an author affiliated with an African university, 90% of the publications had an African university first author and 41% of the papers have CARTA fellows as the first author. Furthermore, 10% of the published papers was authored by more than one CARTA fellow. We also found that 70% of the publications were from fellows affiliated with participating universities in Kenya, Nigeria, and South Africa.

**Table 2:** Summary of characteristics of publications and authors

***Summary characteristics of publications and authors.***	
*Total number of publications*	*806*
*Total number of fellows *	*140*
*Median impact factor of the journals*	*2.10 (IQR: 1.04–2.78).*
*Number of online versions of published papers*	*6308*
*Number of times all papers have been cited*	*5529*
*Number of countries of author affiliation*	*10*
*Number of host institutions*	*9*
*Number of papers with a CARTA fellow as first author*	*318 (41%)*
***Authorship teams***	
*Single-authored publications (%)*	*29 (3.6%)*
*Publications with 2–5 authors (%)*	*434 (61.5%)*
*Publications with ≥ 5 authors (%)*	*246 (34.9%)*
*Mean number of authors per publication*	*5(sd:4)*
***Research approach***	
*Mixed methods*	*6%*
*Qualitative*	*12%*
*Quantitative*	*72%*
*Review*	*10%*

The average number of authors per publication was 5 (SD: 4). The majority of the papers were published by two to five authors (61.5%), and 34.9% of the published articles included more than five authors. Fewer publications (3.6%) had a single author. The median number of publications per fellow per year was 3 (IQR: 2–6). Table 2 also shows that the majority of the publications were quantitative research studies (72%).

### Publication by research area

**Fig. 2:**
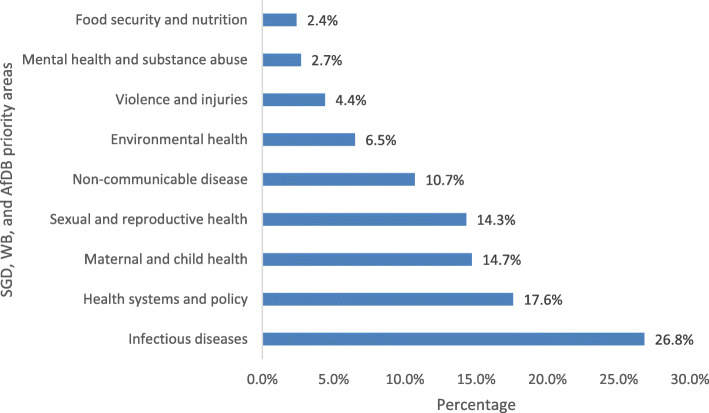
Percentage of CARTA publications by research priority area

About 69% of the 806 published papers fell into the nine priority areas. Figure 2 shows the papers published in the nine priority areas, the highest proportion of the publications were on infectious diseases with at least one out of four articles published (26.8%) in this priority area. This was followed by health systems and policy (17.6%), maternal and child health (14.7%), sexual reproductive health (14.3%), and NCDs (10.7%). A smaller proportion of articles were on contemporary challenges such as environmental health (6.5%), violence and injuries (4.4%), and food security and nutrition (2.4%).

Approximately 28% of the publications in the different health priority areas listed in Table 1 were from studies that targeted females exclusive or a combination of females and males (67.9%) and few focused on males only (4.1%). The few males only publications were predominantly on infectious diseases (31.3%) and sexual and reproductive health (31.3%). By age group, 68.2% of the studies targeted people aged 20 years old and above, about 18.4% of the studies targeted children under 10 years old and 13.5% of the studies targeted adolescents (10–19 year olds). Of note is that mental health and substance abuse publications were only among people aged 20 years old and above, with none among adolescents and children under 10 years old.

#### Infectious diseases

**Fig. 3:**
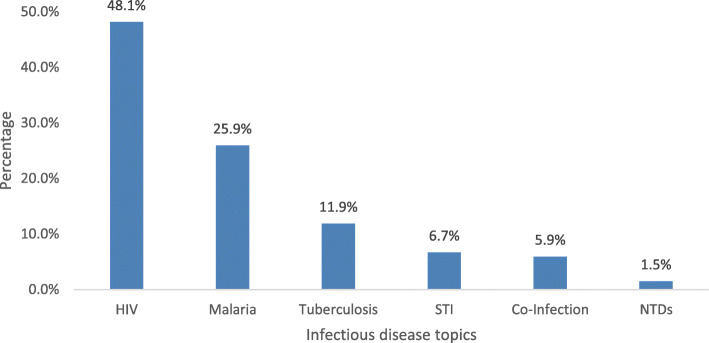
Type of infectious disease topics covered by fellows’ publications

The 135 infectious disease publications focused on prevalent diseases common in Africa, including HIV and AIDS (48.1%), followed by malaria (25.9%), TB (11.9%), and other STIs (6.7%), as shown in Fig. 3. There was minimal research in the areas of co-infections (5.9%) and neglected tropical diseases (NTDs) (1.5%). The majority of the infectious disease publications targeted females and males together (79.6%), followed by females only (15.7%) and males only (4.6%). By age group, most of the studies (67.4%) targeted people aged 20 years old and above, followed by children under 10 years old (18.6%) and for adolescents aged 10 to 19 years old (13.5%).

#### Maternal and child health (MCH)

**Fig. 4:**
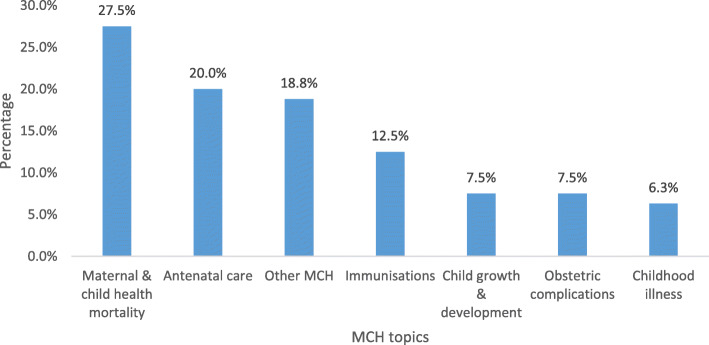
Topical Maternal and child health publication outputs

The publications on MCH comprised of 80 papers constituting 14.7% of the research output. The main topics of focus in this priority area were maternal and child mortality (27.5%), antenatal care (20.0%), and immunisation (12.5%). Obstetric complications, and child growth and development contributed 7.5% to this category, followed by childhood illnesses (6.3%) as shown in Fig. 4. The majority of the publications on MCH targeted females and males together (53.3%), followed by females only (41.9%) and males only (4.8%). By age group most of the studies targeted people aged 20 years old and above (52.4%) followed by children under 10 years old (46.3%) and adolescents aged 10 to 19 years old (1.6%).

#### Sexual and reproductive health (SRH)

**Fig. 5:**
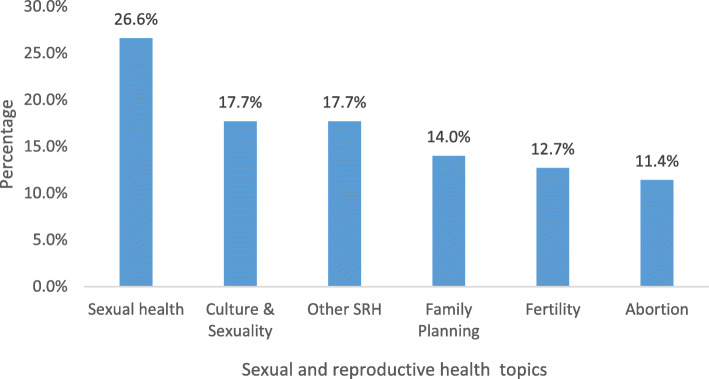
Sexual and reproductive health publications

Overall, 79 publications addressed SRH issues (17.7% of total publications). More than a quarter of the publications on this topic were on adolescent sexual health (26.6%), followed by culture and sexuality (17.7%) and family planning (14.0%). Research on fertility and abortion constituted 12.7 and 11.4%, respectively. Other areas under SRH included risky sexual behaviours and transactional sex (Fig. 5). The majority of the publications on SRH targeted females only (53.9%), followed by females and males together (38.5%), and males only (7.7%). By age group, most of the studies targeted people aged 20 years old and above (64.4%) followed by adolescents at 35.6%.

#### Health systems and policy

The majority of health systems and policy publications (*n* = 97) dealt with health system strengthening (76.0%) followed by the use of mHealth solutions (10.4%) and health economics (8.3%).

#### Non-communicable diseases (NCDs)

Fifty-nine publications (10.7%) of the total publications focused on NCDs. Half of the research output classified under NCDs was on cancers (51.0%), followed by cardiovascular diseases (27.0%), diabetes (16%), and obesity 5.0%. Approximately 5.0% of the publications on NCDs were among children and adolescents while 95% were among people aged 20 years old and above.

#### Violence and injuries

A total of 24 (4.4%) of the total publications were on violence and injuries. Most of these publications were on intimate partner violence (75.0%), and the rest were on bullying (12.5%) and general injuries (12.5%). While similar proportions of publications on violence and injuries were conducted among females only and males and females combined at 47% respectively, about 6% of the studies focused on males only.

#### Environmental health

About 6.5% of the total publications were on environmental health. Most of the publications on environmental health dealt with environmental impact assessment (45%) followed by historical public health challenges such as water and sanitation (37.1%) and indoor air pollution (20%).

#### Funding sources

Finally, about 50% of the published papers had information on additional sources of direct and indirect financial support and we counted at least 160 different sources of support. The most commonly reported sources of additional support were National Institute of Health (12.7%), United States Agency for International Development (4.3%), Wellcome Trust (3.8%), National Research Foundation (3.6%) and the South African Medical Research Council (3.3%).

## Discussion

Building and sustaining research capacity has been advocated as a leading strategy to overcome health disparities in Africa [25]. Our analysis showed a steady increase in the annual number of articles published by CARTA fellows in the 8 years studied, a finding that has been reported elsewhere [27]. The year-on-year increase in publications is likely to be as a result of the corresponding increase in the number of CARTA fellows each year. In 2011 there were 20 fellows in the programme and by 2018, 185 fellows had been admitted in the fellowship. The areas of study were likely a reflection of the increased global response to infectious diseases such as HIV and AIDS, TB and malaria through funding programs such as the Global Fund and the investments by the National Institute of Health and the United States Agency for International Development reported in the fellows' publications. Increased funding in these areas may have influenced the selection of research topics by CARTA fellows. Studies have reported that HIV/AIDS, malaria and tuberculosis (TB) continue to attract the greatest investment from external funders, a trend that influences current research being conducted in sub-Saharan Africa [16, 28].

In our analysis, the subject areas addressed by CARTA PhD fellows are mostly aligned with the SDGs, World Bank and AfDB priority areas, addressing research gaps in infectious diseases, maternal and child health, and sexual and reproductive health. These research areas are in line with the burden of disease and health system priorities in the region. Studies show that 90% of children who die from malaria are from sub-Saharan Africa (SSA), and 72% of deaths in the region are attributed to infectious diseases including HIV/AIDS, TB, and Malaria, and from complications related to pregnancy and childbirth [26].

Despite the increasing number of NCDs including cancers, diabetes and hypertension – that are now co-occurring alongside infectious chronic diseases (such as HIV and TB) [29], findings from this study show less research is conducted in this field. In addition, in the face of high burden of neglected tropical diseases, such as helminth infections, hookworms, and other protozoa amongst the poorest in SSA [30], our analysis showed that while this is being studied, very few papers were found in this area. The same trend was observed in infectious diseases such as hepatitis B and C, despite the substantial burden that these diseases have in Africa [31, 32]. Notably, only a few fellows researched violence and injury. This finding concurred with earlier research indicating that despite violence and injury-related morbidity being at the top of the list of disease burden in Africa, the field remains under-researched [33]. In addition, there were no studies on mental health and substance abuse among children and adolescents; this is in spite of their correlation and burden in the continent [34, 35]. In light of these findings, we recommend that African universities broaden their research priorities in tandem with the change in disease burden taking place in Africa. Contextually focused research would provide appropriate evidence-based information to guide policies and decisions aimed at addressing disease burden and future epidemics in Africa [2].

Our analyses also show that most articles were published by fellows from or affiliated to consortium universities in Kenya, Nigeria, and South Africa. The three countries are also home to five of the nine host institutions in the consortium. This finding is similar to earlier studies that revealed that health research productivity in Africa is skewed, with countries like South Africa, Nigeria, and Kenya contributing more than half of all research papers indexed in PubMed between 2000 and 2014 [17]. This could also be due to differences in economic development and education levels (including epidemiology or public health programmes), which vary widely between and even within countries [11, 26]. Thus, it is paramount to keep developing research capacity in partner institutions with low research output by ensuring an enabling environment for conducting research and training more PhDs in such institutions.

The increase in the number of articles published by CARTA fellows contributes to improved health research output of African academic and research institutions. Whereas externally supported researchers from sub-Saharan Africa have often undertaken postgraduate degrees at institutions in high-income countries [9], CARTA has demonstrated a mechanism to increase PhD training in African institutions. Local enrolment is supplemented by additional PhD training – CARTA’s Joint Advanced Seminars (JAS) to ensure PhD students are internationally competitive and also orients them towards locally relevant research [36]. Such initiatives could be supported by African governments (in respective countries) by prioritising research funding and considering the needs of young researchers in Africa. Only if African governments invest in young researchers will the continent be able to come up with innovations that are relevant to solving the public health problems affecting the population of the region.

### Limitations

We acknowledge some limitations of our study. Although it reports on the number of publications by CARTA fellows, it lacks information on the impact these publications have on policy implementation. This study relied on self-reporting of publications to the CARTA secretariat alone; publication outputs not reported were not included. While we recognise the number of times fellows’ publications have been cited, we did not ascertain how much of it were self-citations. Lastly, CARTA fellows are only a small subset of all PhDs in any institution and this report is not a reflection of the full range of topics studied at the institutions.

## Conclusions

This study demonstrates the full range of topics that CARTA fellows are researching on. The evaluation has shown that investments by CARTA in supporting doctoral training at African training institutions has been beneficial to the region as the programme has provided the PhD fellows sufficient motivation and skills to publish their research in the broad fields of public and population health. There is a need to keep developing research capacity in countries with low research output by training more PhDs in such institutions and by facilitating enabling environments for research. Further and broader exploration of nature and the drivers of public and population health research output remains an imperative to address gaps, promote alignment and sustainability of public health training, research and practice in sub-Saharan Africa.

## Data Availability

The data that support the findings of this study are available from the corresponding author on reasonable request.

## Abbreviations

AAS: African Academy of Science
AESA: Accelerating Excellence in Science in Africa
AfDB: Africa Development Bank
AFRO: African Region
AIDS: Acquired Immunodeficiency Syndrome
APHRC: African Population and Health Research Centre
CARTA: Consortium for Advanced Research Training in Africa
CDC: Centre for Disease Control
DAAD: Deutscher Akademischer Austauschdienst
DELTAS: Developing Excellence in Leadership, Training and Science
HIV: Human Immunodeficiency Virus
IQR: Interquartile Range
MCH: Maternal and Child Health
MDGs: Millennium Development Goals
NCDs: Non-Communicable Diseases
NEPAD: New Partnership for Africa’s Development
NTD: Neglected Tropical Disease
SRH: Sexual and Reproductive Health
SDG: Sustainable Development Goals
TB: Tuberculosis
WB: World Bank
WHO: World Health Organization
